# Supplementary material for: Cost-utility analysis of home blood pressure measurement for screening and diagnosis of hypertension through village health volunteer mechanism in Thailand
Source: PLoS One. 2024 Oct 24;19(10):e0308851. doi: 10.1371/journal.pone.0308851 (PMC11500845; doi:10.1371/journal.pone.0308851)
Supplement: S1 Table — (DOCX) [file pone.0308851.s001.docx]

S1 Table. Parameters Used in the Model after Calibration

| **Parameter** | **Distribution** | **Mean** | **95%CI** | **References** |
| --- | --- | --- | --- | --- |
| **HT incidence** |  |  |  |  |
| Age 35 – 39 years | Beta | 0.004 | 0.00 - 0.004 | [7, 48] |
| Age 40 – 49 years | Beta | 0.015 | 0.012 – 0.018 | [7, 48] |
| Age 50 – 59 years | Beta | 0.019 | 0.016 - 0.022 | [7, 48] |
| Age 60 – 69 years | Beta | 0.027 | 0.021 – 0.033 | [7, 48] |
| Age > 70 years | Beta | 0.034 | 0.021 – 0.047 | [7, 48] |
| **Diagnostic performance** |  |  |  |  |
| Sensitivity of CBPM | Beta | 0.74 | 0.736 – 0.781 | [10] |
| Specificity of CBPM | Beta | 0.79 | 0.749 – 0.831 | [10] |
| Sensitivity of HBPM | Beta | 0.71 | 0.664 – 0.756 | [10] |
| Specificity of HBPM | Beta | 0.82 | 0.794 – 0.846 | [10] |
| Sensitivity of serial1 | Beta | 0.47 |  | [10] |
| Specificity of serial1 | Beta | 0.94 |  | [10] |
| Sensitivity of serial2 | Beta | 0.90 |  | [10] |
| Specificity of serial2 | Beta | 0.58 |  | [10] |
| **Mortality rate** |  |  |  |  |
| Age 35 – 39 years | Beta | 0.003 | 0.003 – 0.003 | [37, 49-51] |
| Age 40 – 49 years | Beta | 0.004 | 0.004 – 0.004 | [37, 49-51] |
| Age 50 – 59 years | Beta | 0.008 | 0.008 – 0.008 | [37, 49-51] |
| Age 60 – 69 years | Beta | 0.016 | 0.016 – 0.016 | [37, 49-51] |
| Age 70 - 79 years | Beta | 0.038 | 0.038 – 0.038 | [37, 49-51] |
| Age 80 – 84 years | Beta | 0.075 | 0.075 – 0.075 | [37, 49-51] |
| Age > 85 years | Beta | 0.151 | 0.150 – 0.151 | [37, 49-51] |
| **HT control rate** |  |  |  |  |
| Controlled HT to uncontrolled HT | Beta | 0.301 | - | Hospital data |
| Uncontrolled HT to controlled HT | Beta | 0.562 | - |  |
| **CVD risk in non-HT people** |  |  |  |  |
| Probability of CVD in non-HT aged 35 - 39 years | Beta | 0.0004 | 0.004 – 0.004 | [52] |
| Probability of CVD in non-HT aged 40 - 49 years | Beta | 0.0004 | 0.0004 – 0.0004 | [52] |
| Probability of CVD in non-HT aged 50 - 59 years | Beta | 0.0013 | 0.0012 – 0.0014 | [52] |
| Probability of CVD in non-HT aged 60 - 69 years | Beta | 0.0023 | 0.0021 – 0.0025 | [52] |
| Probability of CVD in non-HT aged 70 - 79 years | Beta | 0.0044 | 0.0040 – 0.0048 | [52] |
| Probability of CVD in non-HT aged 80 - 84 years | Beta | 0.0081 | 0.0073 – 0.0089 | [52] |
| Probability of CVD in non-HT age > 85 years | Beta | 0.0124 | 0.0112 – 0.0136 | [52] |
| **CVD risk in controlled HT** |  |  |  |  |
| Probability of CVD in controlled HT aged 35 - 39 years | Beta | 0.00148 | 0.001 – 0.002 | [52, 53] |
| Probability of CVD in controlled HT age 40 - 49 years | Beta | 0.00148 | - | [52, 53] |
| Probability of CVD in controlled HT age 50 - 59 years | Beta | 0.005 | - | [52, 53] |
| Probability of CVD in controlled HT age 60 - 69 years | Beta | 0.007 | - | [52, 53] |
| Probability of CVD in controlled HT age 70 - 79 years | Beta | 0.015 | - | [52, 53] |
| Probability of CVD in controlled HT age 80 - 84 years | Beta | 0.032 | - | [52, 53] |
| Probability of CVD in controlled HT age > 85 years | Beta | 0.041 | - | [52, 53] |
| **CVD risk in uncontrolled HT** |  |  |  |  |
| Probability of uncontrolled HT to CVD age 35 - 39 years | Beta | 0.003 | 0.003 – 0.003 | [52, 53] |
| Probability of uncontrolled HT to CVD age 40 - 49 years | Beta | 0.003 | 0.003 – 0.003 | [52, 53] |
| Probability of uncontrolled HT to CVD age 50 - 59 years | Beta | 0.009 | 0.0086 – 0.0094 | [52, 53] |
| Probability of uncontrolled HT to CVD age 60 - 69 years | Beta | 0.013 | 0.0124 – 0.0136 | [52, 53] |
| Probability of uncontrolled HT to CVD age 70 - 79 years | Beta | 0.028 | 0.027 – 0.029 | [52, 53] |
| Probability of uncontrolled HT to CVD age 80 - 84 years | Beta | 0.059 | 0.057 – 0.061 | [52, 53] |
| Probability of uncontrolled HT to CVD age > 85 years | Beta | 0.076 | 0.072 – 0.080 | [52, 53] |
| **HT risk in WCHT** |  |  |  |  |
| Incidence of HT in WCHT age 35-39 years | Beta | 0.004 | 0 – 0.004 | [7, 48] |
| Incidence of HT in WCHT age 40 - 49 years | Beta | 0.015 | 0.012 – 0.018 | [7, 48] |
| Incidence of HT in WCHT age 50 - 59 years | Beta | 0.020 | 0.017 – 0.023 | [7, 48] |
| Incidence of HT in WCHT age 60 - 69 years | Beta | 0.028 | 0.022 – 0.034 | [7, 48] |
| Incidence of HT in WCHT age 70 - 79 years | Beta | 0.034 | 0.021 – 0.047 | [7, 48] |
| **CVD fatality rate** |  |  |  |  |
| Age < 45 years | Beta | 0.007 | 0.006 – 0.008 | [35, 36] |
| Age > 45 years | Beta | 0.09 |  | [35, 36] |
| Screening coverage | Beta | 0.74 | 0.67 – 0.81 | [36] |
| **Annual screening cost** |  |  |  |  |
| Screening with CBPM | Gamma | 76.2 | - | Data collection |
| Screening with HBPM | Gamma | 520.3 | - |  |
| Screening with Serial1 | Gamma | 82.6 | - |  |
| Screening with Serial2 | Gamma | 187.7 | - |  |
| **Annual direct medical cost** |  |  |  |  |
| Acute and subacute stroke | Gamma | 36,305.01 | 34,016.45 – 38,593.57 | [38] |
| Continuous care at OPD for stroke | Gamma | 1,794 | 0 – 3,588 | [41] |
| First year treatment of CAD | Gamma | 228,196.09 | 219,196.09 – 236,822.64 | [42] |
| After first year treatment of CAD | Gamma | 18,255.69 | 17,494.14 – 19,017.24 | [42] |
| Continuous care at OPD for HT | Gamma | 3453.24 | 87.64 – 6,818.84 | [40] |
| **Annual direct non-medical cost in treatment at OPD** |  |  |  |  |
| Controlled HT | Gamma | 655.41 | 0 – 1,310.82 | [39] |
| Uncontrolled HT | Gamma | 1506.9 | 0 – 3,013.8 | [39] |
| CVD | Gamma | 1,899.1 | 0 – 3,798.2 | [39] |
| **Annual direct non-medical cost in treatment at IPD** |  |  |  | [39] |
| CVD | Gamma | 3244.5 | 0 – 6,489 | [39] |
| **Utility** |  |  |  |  |
| CVD | Beta | 0.62 | - | [45, 47] |
| Controlled HT | Beta | 0.954 | 0.949 – 0.959 | [45] |
| Uncontrolled HT | Beta | 0.9 | 0.895 – 0.905 | [45] |

CVD; cardiovascular disease, CAD; coronary artery disease, CPI; consumer price index, HT; hypertension, MHT; masked hypertension, OPD; out-patient department, IPD; inpatient-department, WCHT; white coat hypertension, HBPM; home blood pressure measurement, serial1; additional HBPM among people with clinic blood pressure >140/90 mmHg, serial2; additional HBPM among people with clinic blood pressure <140/90 mmHg

Table 2. Clinical outcomes and cost-utility results based on a societal perspective

| **Results** | **CBPM** | **HBPM**  **(incremental value)** | **Serial1**  **(incremental value)** | **Serial2**  **(incremental value)** |
| --- | --- | --- | --- | --- |
| **Clinical outcomes** |  |  |  |  |
| HT prevalence (%) | 36.21% | 36.21% | 36.19% | 36.22% |
| Incidence of CVD | 19.78% | 19.79% | 19.97% | 19.78% |
| CVD mortality | 13.28% | 13.28% | 13.43% | 13.26% |
| WCHT incidence | 0.763 | 0.748 | 0.426 | 0.798 |
| Probability to be undiagnosed HT | 0.021 | 0.028 | 0.155 | 0.007 |
| **Cost-utility analysis results** |  |  |  |  |
| Total LYs | 39.941 | 39.950 (0.009) | 39.926 (-0.015) | 39.953 (0.012) |
| Total QALYs | 22.156 | 22.152  (- 0.004) | 22.130  (-0.026) | 22.157  (0.001) |
| Total costs | 111,699 | 111,424  (-275) | 78,862  (-32,837) | 119,020  (7,321) |
| Screening cost | 433 | 2,718  (2,285) | 1,106  (673) | 643  (210) |
| Treatment cost | 111,266 | 108,706  (-2,560) | 77,756  (-33,510) | 118,377  (7,111) |
| Incremental cost |  | -275 | -32,837 | 7,321 |
| Incremental QALY |  | -0.004 | -0.026 | 0.001 |
| ICER |  | 68,750 | 1,262,962 | 7,321,000 |

HT; hypertension, CVD; cardiovascular diseases, WCHT; white coat hypertension, LY; life years, QALY; quality adjusted life years, ICER; incremental cost-effectiveness ratio, HBPM; home blood pressure measurement, serial1; additional HBPM among people with clinic blood pressure >140/90 mmHg, serial2; additional HBPM among people with clinic blood pressure <140/90 mmHg
