# Supplementary material for: Cost-utility analysis of home blood pressure measurement for screening and diagnosis of hypertension through village health volunteer mechanism in Thailand
Source: PLoS One. 2024 Oct 24;19(10):e0308851. doi: 10.1371/journal.pone.0308851 (PMC11500845; doi:10.1371/journal.pone.0308851)
Supplement: S1 File — (DOCX) [file pone.0308851.s003.docx]

**Interview questions to estimate the cost of screening**

**Question 1**: What are overall activities for CBPM and HBPM screening?

Part 1: What is demographic characteristics of interviewee such as name, department, and position?

Part 2: What is the flow of the following activities?

- Target group preparation process
- Team and equipment preparation process (responsible members, meeting, medical equipment, document, data management, storage and processing)
- Screening process (community appointment, screening venue, operating steps, roles of the participants, operating period and duration, time consuming)
- Data management process: data collection, data transfer, data sender and receiver
- Follow up suspected case for continuity service process

**Question 2**: What are the activities as an individual staff?

Part 1: What is demographic data of interviewee such as name, department, and position?

Part 2: How is your relevance to CBPM and HBPM screening?

- Target population preparation process
- Team and equipment preparation process
- Screening operation process
- Follow up suspected case for continuity service process

Part 3: What are capital, labor, and material utilizations for CBPM and HBPM screening?

- Capital
  - Office space
  - Rental price
  - Meeting venue
- Labor
  - Daily productivity and time consuming
  - Salary and compensations
  - Honorarium for transportation of participants
- Material
  - Blood pressure measurement device: model, year of purchasing, price
  - Vehicles: model, year of purchasing, price, frequency and duration of usage
  - Office supplies: frequency, duration, amount and price

**Question 3**: What are data from finance and parcel departments?

- Units involving with CBPM and HBPM screening
- Blood pressure measurement equipment: model, date of purchase, warranty period, type of battery, repair history, purchase price
- Vehicle: model, date of purchase, warranty period, type of battery, repair history, purchase price
- Communication equipment: model, date of purchase, warranty period, type of battery, repair history, purchase price
- Computer: model, date of purchase, warranty period, type of battery, repair history, purchase price
- Office supplies
- Salary and compensation of stakeholders
- Training for the team and volunteers
