# Supplementary material for: Cost-utility analysis of home blood pressure measurement for screening and diagnosis of hypertension through village health volunteer mechanism in Thailand
Source: PLoS One. 2024 Oct 24;19(10):e0308851. doi: 10.1371/journal.pone.0308851 (PMC11500845; doi:10.1371/journal.pone.0308851)
Supplement: Attachment — Submitted filename: Editors comments.docx [file pone.0308851.s004.docx]

**Editor’s comments**

**1. Introduction**

The introduction has a lot of inconsistencies, grammatical errors and typos. i.e line 78 and 79; you wrote mm/Hg and line 91; you wrote mmHg.

The paragraph 76-83 is not important as it discusses the specificity and sensitivity. I would much prefer reading results of other studies done on cost-effectiveness of ABPM or HBPM. At least 3 studies discussing the cost effectiveness of ABPM or HBPM or CBPM is necessary

The aim of this study is confusing “this study aimed to evaluate the cost-effectiveness of HBPM as either primary or secondary screening after receiving CBPM compared with CBPM only, a current practice”. Kindly re-formulate the aim in an easy way to understand for the reader

**2.Methodology**

Kindly specify the Study design

Kindly separate ethical consideration from cost and elaborate it alone

Table 1. Parameters used in the model after calibration: This table may be put in the supplementary documents

It is not clear how utility was calculated

Line 138-140:

“The HT incidence and prevalence data were retrieved from the cohort of officers working in

the Electricity Generating Authority of Thailand (EGAT) aged 30 -79 years during 2012-2014 and 2017-2019”

I have a serious concern on the source of the data used in this study. Trusted data should come from a national survey or a census. The electricity generating authority of Thailand (EGT) does not represent the Thailand population. We cannot assume that EGT data can be generalized to the rest of the Thailand population. The use of such data must be highly justified!

In addition, it is not clear how the probabilities of death due to these diseases were calculated (Ischemic stroke, hemorrhagic stroke, CAD, …)

It is also not clear how the transitional probabilities and probability to develop HT among WCHT were calculated

**Costs calculation**:

1.You said you used a societal perspective in the study, direct medical and non-medical costs were included. Yet, in a societal perspective, indirect costs (the loss of income due to absenteeism at work: salary missed when at the hospital or clinic) must be included. And it is not explained how it was calculated. If there were no costs linked to CBPM, kindly explain this

2. Line 159-161: It is not clear at all how the costs of HT screening was estimated based on HT screening related activities by interviewing health officers and VHV in Chachoengsao province during April-December 2019

Table1: Should be the supplementary documents

Statistical analysis is missing: There should be a paragraph to give details of how statistical analysis was performed including the details of cost-effectiveness plane. I will recommend to pull the cost-effectiveness plane to the main document and explain which procedures are cost-effective

**Findings**

The results are not presented in the standard manner according to Consolidated Health Economic Evaluation Reporting Standards (CHEERS). Kindly follow the details of CHEERS below;

<https://www.valueinhealthjournal.com/article/S1098-3015(13)00022-3/fulltext?_returnURL=https%3A%2F%2Flinkinghub.elsevier.com%2Fretrieve%2Fpii%2FS1098301513000223%3Fshowall%3Dtrue>

<http://www.ispor.org/TaskForces/EconomicPubGuidelines.asp>

1. Study parameters: Reports, ranges, references,--- are missing

2. Incremental costs and outcomes: “For each intervention, report mean values for the main categories of estimated costs and outcomes of interest, as well as mean differences between the comparator groups. If applicable, report incremental cost-effectiveness ratios”

3. Characterizing uncertainty: “*Single study-based economic evaluation:* Describe the effects of sampling uncertainty for the estimated incremental cost and incremental effectiveness parameters, together with the impact of methodological assumptions (such as discount rate, study perspective)”.

4. Characterizing heterogeneity: “If applicable, report differences in costs, outcomes, or cost-effectiveness that can be explained by variations between subgroups of patients with different baseline characteristics or other observed variability in effects that are not reducible by more information”.

**Discussion**

Line 248: “Our results indicated that at the societal WTP threshold of 160,000 baht per QALY gained,”. The willingness to pay for Thai peoples could have been discussed in the introduction

**Conclusion**

The conclusion of this study does not support the results of the study
