# Supplementary material for: Cost-utility analysis of home blood pressure measurement for screening and diagnosis of hypertension through village health volunteer mechanism in Thailand
Source: PLoS One. 2024 Oct 24;19(10):e0308851. doi: 10.1371/journal.pone.0308851 (PMC11500845; doi:10.1371/journal.pone.0308851)
Supplement: Attachment — Submitted filename: Response to Reviewers-28Mar24.docx [file pone.0308851.s005.docx]

**Journal Requirements:**

1. When submitting your revision, we need you to address these additional requirements.

Please ensure that your manuscript meets PLOS ONE's style requirements, including those for file naming. The PLOS ONE style templates can be found at 
https://journals.plos.org/plosone/s/file?id=wjVg/PLOSOne_formatting_sample_main_body.pdf and https://journals.plos.org/plosone/s/file?id=ba62/PLOSOne_formatting_sample_title_authors_affiliations.pdf

**Response:** Thank you for your suggestion. We ensure that our manuscript meets PLOS ONE’s style requirements, including those for file naming.

2. Did you know that depositing data in a repository is associated with up to a 25% citation advantage (https://doi.org/10.1371/journal.pone.0230416)? If you’ve not already done so, consider depositing your raw data in a repository to ensure your work is read, appreciated and cited by the largest possible audience. You’ll also earn an Accessible Data icon on your published paper if you deposit your data in any participating repository (https://plos.org/open-science/open-data/#accessible-data).

**Response:** Thank you for your suggestion. All data have been reported in our manuscript.

3. We noticed you have some minor occurrence of overlapping text with the following previous publication(s) among others, which needs to be addressed:

In your revision ensure you cite all your sources (including your own works), and quote or rephrase any duplicated text outside the methods section. Further consideration is dependent on these concerns being addressed.

https://academic.oup.com/ajh/article/28/5/595/2743434?login=false

**Response:** Thank you for pointing this out. We have paraphrased the sentence and put the citation as follows.

*“Thirdly, while prolonged periods of BP measurement may lead to a slight improvement in diagnostic accuracy (52), our study did not fully explore this aspect.”*

https://ecronicon.com/ecemr/pdf/prevalence-and-predictors-of-hypertension-type-2-diabetes-mellitus-comorbidity-among-patients-in-wachemo-university-nigest-elleni-mohammed-memorial-referral-hospital-southern-ethiopia.pdf?

**Response:** We have paraphrased the sentence and put the citation as follows.

*“The presence of HT can substantially elevate the risk of various cardiovascular diseases (CVD), including coronary heart diseases (CHD), congestive heart failure (CHF) and peripheral arterial diseases (2). Moreover, it is a major contributor to the incidence of both ischemic and hemorrhagic stroke, as well as renal failure (2).”*

4. Thank you for stating in your Funding Statement:

This work was supported by funding from Mahidol University and the International Decision Support Initiative (iDSI) through the doctoral study in Mahidol University Health Technology Assessment (MUHTA) Graduate Program. This work was produced as part of the iDSI (www.idsihealth.org), which supports countries to get the best value for money from health spending. iDSI receives funding support from the Bill & Melinda Gates Foundation and the UK Department for International Development (Grant No. OPP1087363). The funders had no role in the study design, data collection and analysis, decision to publish, or preparation of the manuscript.

Please provide an amended statement that declares all the funding or sources of support (whether external or internal to your organization) received during this study, as detailed online in our guide for authors at http://journals.plos.org/plosone/s/submit-now.  Please also include the statement “There was no additional external funding received for this study.” in your updated Funding Statement. Please include your amended Funding Statement within your cover letter. We will change the online submission form on your behalf.

**Response:** Thank you for your helpful suggestions. We have provided an amended statement that declares all the funding or sources of support (whether external or internal to our organization) received during this study. We have already included the statement “There was no additional external funding received for this study” in our updated Funding Statement within our cover letter.

5. We note that your Data Availability Statement is currently as follows: All relevant data are within the manuscript and its Supporting Information files.

Please confirm at this time whether or not your submission contains all raw data required to replicate the results of your study. Authors must share the “minimal data set” for their submission. PLOS defines the minimal data set to consist of the data required to replicate all study findings reported in the article, as well as related metadata and methods (https://journals.plos.org/plosone/s/data-availability#loc-minimal-data-set-definition).

For example, authors should submit the following data:

The values behind the means, standard deviations and other measures reported;
- The values used to build graphs;
- The points extracted from images for analysis.

Authors do not need to submit their entire data set if only a portion of the data was used in the reported study.

If your submission does not contain these data, please either upload them as Supporting Information files or deposit them to a stable, public repository and provide us with the relevant URLs, DOIs, or accession numbers. For a list of recommended repositories, please see https://journals.plos.org/plosone/s/recommended-repositories.

If there are ethical or legal restrictions on sharing a de-identified data set, please explain them in detail (e.g., data contain potentially sensitive information, data are owned by a third-party organization, etc.) and who has imposed them (e.g., an ethics committee). Please also provide contact information for a data access committee, ethics committee, or other institutional body to which data requests may be sent. If data are owned by a third party, please indicate how others may request data access.

**Response:** Thank you for your helpful suggestions. Data cannot be shared publicly because there are ethical restrictions on publicly sharing a data set. Data are available from the Research Ethics Protection Unit, the Faculty of Dentistry/Faculty of Pharmacy, Mahidol University (contact via Dental Simulation and Research Building, 5th Floor Mahidol University Faculty of Dentistry, 6, Yothi Road, Ratchathewi, Bangkok, 10400 or +662-200-7622) for researchers who meet the criteria for access to confidential data.

**Additional Editor Comments**

1. **Introduction**

The introduction has a lot of inconsistencies, grammatical errors and typos. i.e line 78 and 79; you wrote mm/Hg and line 91; you wrote mmHg.

**Response:** Thank you for your helpful suggestion. We have already corrected the grammatical errors and typos in the introduction section as suggested.

The paragraph 76-83 is not important as it discusses the specificity and sensitivity. I would much prefer reading results of other studies done on cost-effectiveness of ABPM or HBPM. At least 3 studies discussing the cost effectiveness of ABPM or HBPM or CBPM is necessary

**Response:** Thank you for your great suggestion. As suggested, we have already revised the sentences as follows (Introduction, page 4, line 78-86).

*“According to a recent systematic review by Wang et al. 2003 (22), four studies compared HBPM to CBPM. Two of these studies focused on HT management (23, 24), while the other two were for diagnosis purpose (25, 26) However, the latter two studies were cost analysis studies that demonstrated medical cost savings in screening with HBPM. Furthermore, the cost-effectiveness study by Lovibond et al. compared screening with ABPM and HBPM to CBPM. The study concluded that screening with ABPM was the most cost-effective strategy across all age groups (50, 60, 70, and 75 years) (27). Additionally, in younger age groups, ABPM yielded greater cost savings but was associated with a slight reduction in quality adjusted life years (QALYs); nevertheless, it remained the most cost-effective option (27).”*

The aim of this study is confusing “this study aimed to evaluate the cost-effectiveness of HBPM as either primary or secondary screening after receiving CBPM compared with CBPM only, a current practice”. Kindly re-formulate the aim in an easy way to understand for the reader

**Response:** Thank you for your helpful suggestions. We have already revised the aim of this study as follows (Introduction, page 4, line 98-99).

*“Therefore, the objective of this study was to assess the cost-effectiveness of BP screening strategies in Thailand.”*

2. **Methodology**

Kindly specify the Study design

**Response:** Thank you for your very helpful suggestion. We have already put sub-heading “Study design” in the Materials and Methods section (Study design, page 5, line 103-109).

*“We conducted a cost-utility analysis using a Markov model to compare costs and health outcomes of BP screening strategies during lifetime horizon with one-year cycle length. length. Health outcomes were life years (LYs) and QALYs, the multiplication of LYs and utility score. The analysis was performed based on a societal perspective, considering both future costs and health outcomes, which were discounted at a rate of 3% per year as recommended by the Thai HTA guidelines (31).”*

Kindly separate ethical consideration from cost and elaborate it alone

**Response:** Thank you for your very useful suggestions. We have separated ethical consideration from cost and elaborate it alone under sub-heading “Ethics approval” (page 5, line 114-117) as below.

*“The ethical approval of this study was granted by the Faculty of Dentistry/Faculty of Pharmacy, Mahidol University. The written informed consent was obtained from the VHV and local officers who participated in the interviews for data collection on costs.”*

Table 1. Parameters used in the model after calibration: This table may be put in the supplementary documents

**Response:** Thank you for your helpful suggestion. We have already put Table 1 in the supporting information as “S1 Table. Parameters Used in the Model after Calibration”.

It is not clear how utility was calculated
Line 138-140:

**Response:** Thank you for raising this point. We have added the sentence to explain how utility was calculated under “Utility” sub-heading (page 9, line 207-209).

*“The proportion of stroke cases in all CVD cases was 0.64, while the proportion of CAD in all CVD was 0.36 (47). Therefore, the overall utility of CVD was calculated as (0.55 x 0.64) + (0.75 x 0.36) = 0.62.”*

“The HT incidence and prevalence data were retrieved from the cohort of officers working in
the Electricity Generating Authority of Thailand (EGAT) aged 30 -79 years during 2012-2014 and 2017-2019”

I have a serious concern on the source of the data used in this study. Trusted data should come from a national survey or a census. The electricity generating authority of Thailand (EGT) does not represent the Thailand population. We cannot assume that EGT data can be generalized to the rest of the Thailand population. The use of such data must be highly justified!

**Response:** Thank you for raising this point. We have added more explanations on EGAT study under Model input parameter sub-heading as follows (page 7, line 150-155).

*“The HT incidence and prevalence data were obtained from the cohort of 2,235 officers aged 30 -79 years employed by the Electricity Generating Authority of Thailand (EGAT) during two periods: 2012-2014 and 2017-2019. The EGAT study represents one of the largest longitudinal cardiovascular cohort studies in the Thai population. Participants with a wide range of socio-demographic backgrounds located in Bangkok and three different sites in Western and Northern Thailand were randomly enrolled in this study.”*

In addition, we also have added the sentences in Discussion part as one of the limitations in this study as follows (page 17, line 379-386).

*“Lastly, our study utilized data from the EGAT cohort, which is one of the largest longitudinal cardiovascular cohort studies in the Thai population. The HT incidence estimated from this cohort might not perfectly represent the entire country, as the socio-economic status of EGAT employees differed from that of many economically disadvantaged individuals in Thailand. Additionally, the ‘healthy worker effect’ might influence baseline BP profiles from the EGAT cohort, further limiting the generalizability of our results (55). Nevertheless, the data from EGAT cohort provide the best available evidence for Thai population with HT.”*

In addition, it is not clear how the probabilities of death due to these diseases were calculated (Ischemic stroke, hemorrhagic stroke, CAD, …)

**Response:** Thank you for your raising this point. The probabilities of death due to hemorrhagic stroke, ischemic stroke, and CAD were obtained from published literature reviews. We have added the sentence as follows (page 7, line 159-162).

*“In this model, CVD consisted of hemorrhagic stroke, ischemic stroke, and CAD. The prognosis of CVD varied depending on the specific disease and could be classified as acute or chronic. The probabilities of death due to hemorrhagic stroke, ischemic stroke, and CAD were borrowed from published literature reviews (32-35).”*

It is also not clear how the transitional probabilities and probability to develop HT among WCHT were calculated

**Response:** Thank you for pointing this out. We have added the sentence to explain how the probability to develop HT among WCHT individuals was calculated as follows (page 8, line 172-174).

*“The probability of developing HT among individuals with WCHT was assumed to be equal to the incidence of HT among the general population.”*

**Costs calculation:**

1. You said you used a societal perspective in the study, direct medical and non-medical costs were included. Yet, in a societal perspective, indirect costs (the loss of income due to absenteeism at work: salary missed when at the hospital or clinic) must be included. And it is not explained how it was calculated. If there were no costs linked to CBPM, kindly explain this
   **Response:** Thank you for your helpful suggestions. In our cost-utility analysis, to prevent double counting, we excluded indirect costs i.e., lost income due to absenteeism at work or foregone wages while attending hospital or clinic appointments. However, we did incorporate the loss of income for caregivers, categorizing it under direct non-medical costs for this study. We have added more explanations under “Cost” sub-heading (page 8, line 180-183) as follows.

*“To avoid double counting in our cost-utility analysis, indirect costs such as lost income due to absenteeism at work or foregone wages while attending hospital or clinic appointments were excluded. However, we did incorporate the loss of income for caregivers, classifying it as direct non-medical costs in this study.”*

2. Line 159-161: It is not clear at all how the costs of HT screening was estimated based on HT screening related activities by interviewing health officers and VHV in Chachoengsao province during April-December 2019

**Response:** Thank you for your helpful suggestions. We have added more explanation how the cost of HT screening was estimated under “Cost” sub-heading (page 8, line 186-194) as follows.

*“Cost of HT screening was estimated by gathering data on HT screening-related activities through interviews with health officers and VHV in Chachoengsao province during April-December 2019. The cost among VHV was estimated based on the duration and frequency of their roles, which included tasks such as preparing lists of targets, providing BP screening with instructions, ensuring coverage of BP screening through home visits, recording BP results, and transmitting the results to primary care units. The cost of health officers was determined by considering the frequency and workload of doctors and nurses involved in HT diagnosis in the extended outpatient department in the communities.”*

Table1: Should be the supplementary documents

**Response:** Thank you for your helpful suggestion. We have already put Table 1 in the supporting information as “S1 Table. Parameters Used in the Model after Calibration”.

Statistical analysis is missing: There should be a paragraph to give details of how statistical analysis was performed including the details of cost-effectiveness plane. I will recommend to pull the cost-effectiveness plane to the main document and explain which procedures are cost-effective

**Response:** Thank you for your helpful suggestions. We have added “Statistical analysis” sub-heading on page 10-11 (line 234-250) to give more details of how statistical analysis was performed including the details of cost-effectiveness plane as follows.

*“Statistical analysis was performed to evaluate the uncertainty surrounding the estimated ICERs and to determine the cost-effectiveness of the interventions using the Microsoft Excel 2019 (Microsoft, WA, USA). One-way sensitivity analysis was conducted to investigate the uncertainty associated with each parameter and the results were visually represented using Tornado diagrams. These diagrams provide a clear illustration of the impact of varying individual parameters on the analysis results. Additionally, probabilistic sensitivity analysis (PSA) was employed to estimate parameter uncertainties surrounding the ICER using Monte Carlo simulation, wherein parameters were simultaneously and randomly selected about 1000 times based on their probability distributions. The results were demonstrated as the cost-effectiveness planes and cost-effectiveness acceptability curves (CEACs). The cost-effectiveness plane consists of a scatter plot with the x-axis representing the difference in costs, and the y-axis depicting the difference in QALYs between studied interventions and comparator. Moreover, CEACs are a graphical representation to present the probability that an intervention is cost-effective across a range of WTP thresholds. At the Thai societal WTP of 160,000 baht per QALY gained, the CEAC will indicate the probability that the intervention is cost-effective compared with the comparator.”*

3. **Findings**
   The results are not presented in the standard manner according to Consolidated Health Economic Evaluation Reporting Standards (CHEERS). Kindly follow the details of CHEERS below;
   https://www.valueinhealthjournal.com/article/S1098-3015(13)00022-3/fulltext?_returnURL=https%3A%2F%2Flinkinghub.elsevier.com%2Fretrieve%2Fpii%2FS1098301513000223%3Fshowall%3Dtrue
   <http://www.ispor.org/TaskForces/EconomicPubGuidelines.asp>
1. Study parameters: Reports, ranges, references,--- are missing

**Response:** Thank you for your helpful suggestions. “S1 Table” included in the Supplementary Information, demonstrates all input parameters used in this study along with their mean values, standard errors and corresponding references.

2. Incremental costs and outcomes: “For each intervention, report mean values for the main categories of estimated costs and outcomes of interest, as well as mean differences between the comparator groups. If applicable, report incremental cost-effectiveness ratios”

**Response:** Thank you for your great suggestions. We have already revised Table 1 including mean values for estimated costs and health outcomes for each intervention, mean differences between BP screening strategies and the comparator (CBPM), as well as incremental cost-effectiveness ratios.

3. Characterizing uncertainty: “Single study-based economic evaluation: Describe the effects of sampling uncertainty for the estimated incremental cost and incremental effectiveness parameters, together with the impact of methodological assumptions (such as discount rate, study perspective)”.

**Response:** Thank you for your helpful suggestions. We have described the effects of sampling uncertainty for the estimated incremental cost and incremental effectiveness parameters, along with the impact of methodological assumptions in the Uncertainty analysis results as follows (page 13, line 274-286).

*“The results of one-way sensitivity analysis revealed that the incidence rate of HT among individuals aged 40-49 years was the most sensitive factor affecting the ICER of HBPM, Serial1 and Serial2. The ICER of Serial2 was most sensitive to the sensitivity of CBPM, the incidence rate of uncontrolled HT leading to CVD among individuals aged 40-49 years, and the incidence rate of HT among individuals aged 50-59 years from a societal perspective (Fig 2). To address the effect of sampling uncertainty on estimated incremental cost and incremental effectiveness parameters using Monte Carlo simulation in our single study-based economic evaluation, a cost-effectiveness plane demonstrated that most simulations of HBPM had higher costs and lower QALY, while Serial1 had substantially lower costs and less QALYs (Fig 3). In contrast, Serial2 was associated with higher costs and higher QALY. Additionally, the cost-effectiveness acceptability curves indicated that Serial1 had a 100% probability of being cost-effective compared with other BP screening modalities at the Thai societal WTP threshold of 160,000 baht per QALY gained (Fig 4).”*

4. Characterizing heterogeneity: “If applicable, report differences in costs, outcomes, or cost-effectiveness that can be explained by variations between subgroups of patients with different baseline characteristics or other observed variability in effects that are not reducible by more information”.

**Response:** Thank you for your helpful suggestions. We have reported the differences in costs, outcomes and cost-effectiveness analysis results of individuals being screened by CBPM and classified into two sub-groups: those with high BP, i.e., clinic BP ≥140/90 mmHg (Serial1), and those without high BP i.e., clinic BP <140/90 mmHg (Serial2), followed by HBPM, as shown in Table 1 (page 12).

**Discussion**
Line 248: “Our results indicated that at the societal WTP threshold of 160,000 baht per QALY gained,”. The willingness to pay for Thai peoples could have been discussed in the introduction

**Response:** Thank you for your great suggestion. We have already added the sentences related to the Thai societal WTP threshold in the introduction section as follows (Introduction, page 4, 94-98).

*“Recently, according to Thai Health Technology Assessment (HTA) Guidelines, the societal willingness to pay (WTP) threshold in Thailand has been set at 160,000 baht per QALY gained (29, 30). This implies that a BP screening strategy with an incremental cost-effectiveness ratio (ICER) below this threshold is considered a cost-effective option and may be considered for inclusion as a national BP screening policy”*

**Conclusion**
The conclusion of this study does not support the results of the study

**Response:** Thank you for your helpful suggestions. We have revised the conclusion as follows (page 17-18, line 389-399).

*“When compared with CBPM within the context of a VHV-based BP screening campaign in Thailand for a population aged 35 years, our study revealed that Serial1 yielded an incremental cost saving of 32,278 baht and an incremental QALY loss of 0.0271. At the societal willingness-to-pay (WTP) threshold of 160,000 baht per QALY gained, the cost saving associated with Serial1 outweighed the QALY loss. On the other hand, Serial2 was not deemed a cost-effective option due to its much higher costs and minimal gains in QALYs. Moreover, HBPM was dominated by CBPM due to its higher costs and lower QALYs. Therefore, based on our findings, it is recommended that Serial1 be implemented as the preferred BP screening option in Thailand. This evidence-informed policy information could be invaluable for policymakers in making rational resource allocation decisions for BP screening through the VHV mechanism in Thailand and similar settings.”*

**Reviewers' comments:**

**Reviewer #1**
Overall, this is a well performed analysis and results provide useful information in the context of middle-income countries. However, it is interesting to note that the cost of treatment of "Serial 1" is distinctly low (77,756 Baht) compared to other strategies (around 108-118,000 Baht) which is quite surprising given that one would expect little differences in hypertension treatment regardless of which method is used for detection or diagnosis. Please explain.

**Response:** Thank you for your helpful suggestions. We have added more explanation below as well as in Discussion part (page 15, line 327-341)

*“In terms of total costs, it was noteworthy to mention that more than 99% of total costs in this study were attributed to treatment costs, encompassing both direct medical and non-medical costs. Although the screening cost of Serial1 was almost the same as that of CBPM, it incurred significantly lower treatment costs* *compared to CBPM, HBPM, and Serial2. Specifically, the savings in direct medical and non-medical costs accounted for 20,876 and 11,405 baht, respectively. These significant cost reductions emphasized the potential cost-saving benefits of Serial1 in comparison to CBPM. Moreover, the Serial1 option yielded the lowest WCHT incidence, thereby potentially reducing unnecessary cost associated with WCHT, which represented a major part of treatment costs. The ability of Serial1 to correctly diagnose WCHT made it the preferred strategy for BP screening, especially in younger adults with a lower prevalence of HT, as recommended by the 2019 Thai Guideline for HT management (10). Although MHT may theoretically lead to more clinically harmful, regular annual BP screening can reduce its prevalence, resulting in a lower incidence and mortality of CVD. Thus, the implementation of Serial1 as a screening modality can potentially mitigate the burden of HT related complications and associated healthcare costs.”*

**Reviewer #2**

The authors present a study comparing three different home blood pressure monitoring strategies (1. Home BP monitoring for all - called HBPM, 2. home monitoring for those with high clinic BP - called serial1, 3. home monitoring for those without high clinic BP - called serial2) in comparison to usual care clinic BP measurement in a Thai community setting. The authors have not followed the CHEERS 2022 reporting guidelines for economic evaluations https://www.equator-network.org/reporting-guidelines/cheers/ which means much of the necessary information is missing.

**Response:** Thank you for your great suggestions. We have revised and followed the CHEER 2022 reporting guidelines for economic evaluation. Please see the attached CHEER checklist of our study.

There are grammatical errors throughout, and this journal does not provide copyediting.

**Response:** Thank you for your helpful suggestions. Grammatical errors throughout the manuscript has been already checked by a professional English editor.

They report underlying data are all available in-text, but the interview data is not available, only the summary statistics in the parameters table. What interview questions were used to elicit these reported costs? Are they reflecting the program running costs?

**Response:** Thank you for your helpful suggestions. Please see the attached interview questions for collecting cost data in our study. Yes, these questions reflected the program running costs.

The model validation is not described clearly - only that it was done by experts.

**Response:** Thank you for your helpful suggestions. We have added more explanations on model validation under Model validation and calibration as follows (page 9-10, line 213-227)

*“Face validity was conducted by a panel of experts including cardiologists, neurologists, internal medicine specialists, biostatisticians, health economists, and clinical epidemiologists and health technology assessment experts. Their roles were to assess and validate the model structure, input parameters and data sources used in this study. Moreover, the criterion validity involved comparing the HT prevalence estimated by the model among individuals aged 35-79 years with the actual prevalence reported in the 5th NHES in 2014 (4) (Fig 2).*

*The model aimed to replicate the actual population composition, including both patients and non-patients, by starting with a population that includes individuals with and without HT or CVD. This approach allowed for a comprehensive assessment of the model's performance in reflecting real-world scenarios. For model calibration, parameters such as age-specific incidence of HT in the Thai population and mortality rates from non-CVD cases were selected due to their association with high uncertainty. Following model calibration, HT prevalence within age group of 45-59, 60-69, and 70-79 years in the model was better fitted with those reported in the 5th NHES (4).”*

I found the naming convention of "serial1" and "serial2" unintuitive to interpret when looking at the results in tables and figures. Consider renaming or adding a footnote to clarify which is HBPM in people with high clinic BP and which is HBPM in people without high clinic BP.

**Response:** Thank you for your great suggestions. We have already added a footnote to clarify that Serial1 is CBPM followed by HBPM among people with clinic blood pressure ≥140/90 mmHg and Serial2 is CBPM followed by HBPM among people with clinic blood pressure <140/90 mmHg in tables and figures.

Consider using better contrast in the Figures so they are still readable with greyscale/black and white printing.

**Response:** Thank you for your very helpful suggestions. We have improved the contrast in all figures to ensure readability, especially for greyscale or black and white printing.

Figure 3 suggests there is substantial overlap in the ICER estimates generated by the PSA - consider how this influences your certainty in interpreting the results and drawing the conclusion.

**Response:** Thank you for your helpful suggestions. After we have re-run the analysis, Figure 3 is now changed to Figure 4 representing that there is no substantial overlap in the ICER estimates generated by the PSA.

While revising your submission, please upload your figure files to the Preflight Analysis and Conversion Engine (PACE) digital diagnostic tool, <https://pacev2.apexcovantage.com/>. PACE helps ensure that figures meet PLOS requirements. To use PACE, you must first register as a user. Registration is free. Then, login and navigate to the UPLOAD tab, where you will find detailed instructions on how to use the tool. If you encounter any issues or have any questions when using PACE, please email PLOS at [figures@plos.org](mailto:figures@plos.org). Please note that Supporting Information files do not need this step.

**Response:** Thank you for your helpful suggestions. We have already revised figure files and uploaded figures files PACE as suggested.
